# Antisense Gapmers with LNA-Wings and (*S*)-5′-*C*-Aminopropyl-2′-arabinofluoro-nucleosides Could Efficiently Suppress the Expression of *KNTC2*

**DOI:** 10.3390/molecules27217384

**Published:** 2022-10-30

**Authors:** Yujun Zhou, Shuichi Sakamoto, Yoshihito Ueno

**Affiliations:** 1 United Graduate School of Agricultural Science, Gifu University, 1-1 Yanagido, Gifu 501-1193, Japan; 2 Institute of Microbial Chemistry (BIKAKEN) Numazu Branch, Microbial Chemistry Research Foundation, 18-24 Miyamoto, Numazu 410-0301, Japan; 3 Faculty of Applied Biological Sciences, Gifu University, 1-1 Yanagido, Gifu 501-1193, Japan; 4 Graduate School of Natural Science and Technology, Gifu University, 1-1 Yanagido, Gifu 501-1193, Japan

**Keywords:** antisense oligonucleotides, 5′-aminopropyl group, 2′-arabinofluoro group, RNase H, phosphorothioate, locked nucleic acid, cancer, KNTC2

## Abstract

Previously reported (*S*)-5′-*C*-aminopropyl-2′-arabinofluoro-thymidine (**5ara-T**) and newly synthesized (*S*)-5′-*C*-aminopropyl-2′-arabinofluoro-5-methyl-cytidine (**5ara-^Me^C**) analogs were incorporated into a series of antisense gapmers containing multiple phosphorothioate (PS) linkages and locked nucleic acids (LNAs) in their wing regions. The functional properties of the gapmers were further evaluated in vitro. Compared with the positive control, for the LNA-wing full PS gapmer without **5ara** modification, it was revealed that each gapmer could have a high affinity and be thermally stable under biological conditions. Although the cleavage pattern was obviously changed; gapmers with **5ara** modification could still efficiently activate *E. coli* RNase H1. In addition, incorporating one **5ara** modification into the two phosphodiester linkages could reverse the destabilization in enzymatic hydrolysis caused by fewer PS linkages. In vitro cellular experiments were also performed, and the Lipofectamine^®^ 2000 (LFA)+ group showed relatively higher antisense activity than the LFA-free group. KN5ara-10, which contains fewer PS linkages, showed similar or slightly better antisense activity than the corresponding full PS-modified KN5ara-3. Hence, KN5ara-10 may be the most promising candidate for *KNTC2*-targeted cancer therapy.

## 1. Introduction

Antisense oligonucleotides (ASOs), composed of approximately 20-bp DNA-like nucleotides, are classified as a kind of mRNA-targeted oligonucleotide therapeutics [1]. Since the first ASO therapeutic, fomivirsen, was approved by the U.S. Food and Drug Administration (FDA) in 1998, nine ASO therapeutics have been approved [2,3,4,5,6,7,8,9,10]. There is no doubt that further research on ASOs will be developed. ASOs can be divided into the following two types depending on the antisense mechanism: the ribonuclease H (RNase H)-dependent type and the splice-switching type [11,12,13]. Both types of ASOs need to be taken up into cytoplasm or nucleoplasm for binding with the targeted mRNAs or pre-mRNAs, which would raise several challenges for the clinical application of ASOs. For example, nucleotides experience difficulty passing through cellular or nuclear membranes because of the negative charges on their phosphodiester (PO) linkages. Moreover, natural DNA strands are quickly degraded via nuclease-mediated hydrolysis inside the plasm, resulting in low pharmacological effects. To overcome these challenges, chemically modified nucleosides have been developed and utilized in approved ASOs.

Phosphorothioate (PS) linkages, which contain sulphur substitutions of non-bridging oxygens at each PO linkage, are the most popular chemical modification to improve the properties of approved ASOs [3,4,5,6,7,8,9,10]. Due to the high degree of similarity between sulfur and oxygen atoms, PS linkages would not obstruct the recognition of ASO/RNA duplexes by RNase H and might even promote the RNase H-mediated cleavage mechanism [14]. PS linkages also help to stabilize ASOs from nucleolytic degradation. In addition, the anionic sulfur in PS linkages could tightly interact with multiple proteins by electrostatic and hydrophobic interactions [15]. Therefore, the application of PS linkages could enhance the membrane permeability of ASOs via binding to membrane proteins. However, the presence of apoptosis of off-target cells, relating to cytotoxicity, was reported in some cases, possibly due to unfavorable interactions between PS linkages and paraspeckle proteins [15]. As general strategies, coordination with other chemical modifications and reducing the content of PS linkages might be beneficial for developing more effective PS-ASOs with less side effects [16,17,18,19,20].

In previous studies, our group designed and synthesized various chemically modified nucleoside analogs containing an aminoalkyl sidechain, which is expected to strengthen nuclease resistance and improve the intracellular internalization of oligonucleotides [21,22,23,24,25,26,27]. As a result of evaluating (*S*)-5ʹ-*C*-aminopropyl- (**5deo**) and (*S*)-5ʹ-*C*-aminopropyl-2ʹ-arabinofluoro- (**5ara**) modified ASOs, it was revealed that ASOs with several **5ara** modifications could significantly enhance the nuclease resistance and show a superior ability to activate the RNase H-dependent antisense mechanism compared with the **5deo**-modified ones, although the cleavage of the complementary RNA strands would be obviously impeded compared with the natural DNAs [27]. 

In this study, we focus on a special formation, named “gapmer”, composed of chemically modified wing regions for high RNA-binding affinity and robust nuclease resistance, and a DNA-based gap region for efficient RNase H-dependent antisense mechanism [3,6,7].

We also focus on locked nucleic acids (LNAs), a class of chemically modified nucleosides, which contain a methylene bridge connecting the 2′ oxygen and 4′ carbon of nucleoside [28,29]. LNA demonstrated high RNA-binding affinity because it locks the ribose conformation into a C3′-endo type, which is beneficial for the A-type formation of ASO/RNA duplex, while the incorporation of LNAs inside oligonucleotides might suppress the recognition of RNase H. Therefore, LNA is often used in the wing regions of gapmers but not in the gap regions. However, even superior properties have been proved in the laboratory level, due to a lack of clinical knowledge, as there are not any cases yet approving LNA-containing ASOs. In this study, an LNA-wing antisense gapmer was selected as an excellent positive control, and the further improvements resulting from the collaboration with **5ara** modification were evaluated, as well as the expedition of LNA into clinical usage.

**Figure 1:**
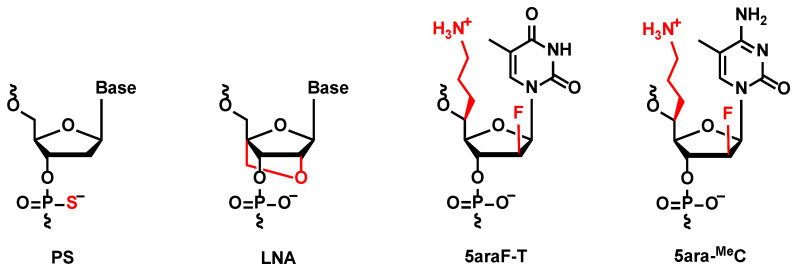
Chemical structure of phosphorothioate (PS), locked nucleic acid (LNA), (*S*)-5ʹ-*C*-aminopropyl-2ʹ-arabinofluoro-thymidine (**5ara-T**), and (*S*)-5ʹ-*C*-aminopropyl-2ʹ-arabinofluoro-5-methyl-cytidine (**5ara-^Me^C**).

The *KNTC2* (Kinetochore associated 2) gene, referred as to *KNTC2*, was selected to be the target gene in this study, which is known to be highly expressed in various cancer cells. Selective knockdown of *KNTC2* was reported as a promising treatment for cancers [30,31,32]. Based on previous findings, we designed a series of *KNTC2*-targeted antisense gapmers with LNA-wings and **5ara** modifications (described as “KN5ara gapmers”) in this study. Each structure of these chemical modifications is shown in Figure 1. The following sections outline the synthesis of KN5ara gapmers by incorporating the reported (*S*)-5ʹ-*C*-aminopropyl-2ʹ-arabinofluoro-thymidine (**5ara-T**) and newly synthesized (*S*)-5ʹ-*C*-aminopropyl-2ʹ-arabinofluoro-5-methyl-cytidine (**5ara-^Me^C**) analogs, followed by the in vitro evaluations of their functional properties.

## 2. Results and Discussion

### 2.1. Oligonucleotide Synthesis

The synthesis of phosphoramidite corresponding to **5ara-^Me^C** is shown in Scheme S1 in the Supporting Information. The modified nucleoside analogs **5ara-T** and **5ara-^Me^C** were incorporated into a series of LNA-wing antisense gapmers utilizing a DNA/RNA synthesizer via the solid-phase phosphoramidite method. After synthesis, to prevent the additional reaction of acrylonitrile with 5ʹ-*C*-aminopropyl groups, the controlled-pore glass (CPG) beads were treated with 10% dimethylamine in MeCN at room temperature for 5 min, followed by rinsing with MeCN to selectively remove cyanoethyl groups. The gapmers were then cleaved from CPG beads and deprotected by treatment with a concentrated NH_3_ solution for 12 h at 55 °C. The corresponding RNA oligomers used in this study were prepared with a DNA/RNA synthesizer. After synthesis, in contrast to antisense gapmers, the RNA oligomers were cleaved from CPG beads and deprotected by treatment with concentrated NH_3_ solution/40% methylamine (1:1, *v*/*v*) for 10 min at 65 °C. Then, 2ʹ-*O*-TBDMS groups in RNA oligomers were removed using Et_3_N∙3HF (125 µL) in DMSO (100 µL) for 1.5 h at 65 °C. The reaction was quenched with a 0.1 M TEAA buffer (pH 7.0) and the mixture was desalted using a Sep-Pak C18 cartridge. The modified antisense gapmers and RNA oligomers were finally purified by 20% denaturing polyacrylamide gel electrophoresis (PAGE) containing 7 M urea. The sequences of the oligonucleotides used in this study are shown in Table 1 and Table S1 (Supplementary Material).

### 2.2. RNA-Binding Affinity

The accurate binding of ASOs to target mRNA is necessary for RNase H recognition and the following antisense mechanism. As reported before, although PS linkages negatively affected thermal stability, LNAs could significantly increase RNA binding affinity and a single (*S*)-5ʹ-*C*-Aminopropyl-2ʹ-arabinofluoro modification caused few changes to the 50% melting temperature (*T*_m_) values of ASO/RNA duplexes [27,28,29]. Therefore, we hypothesized that the series of KN5ara gapmers would have sufficient RNA binding affinity for therapeutic application. In this study, each KN5ara gapmer was mixed in the same volume of cRNA-1, and then annealed to form ASO/RNA duplexes. Temperature-induced melting was measured by ultraviolet (UV) spectroscopy in a 10 mM sodium phosphate buffer (pH 7.0) containing 100 mM NaCl, and *T*_m_ values were obtained from melting curves using the standard method. Each Δ*T*_m_ was calculated from [*T*_m_ (duplex containing each KN5ara gapmers) − *T*_m_ (duplex containing KN-pos)].

As shown in Table 1, all KN5ara gapmers maintained a stable duplex structure with complementary RNA strands at 37 °C. The incorporation of **5ara**-modified analogs in the gap region showed moderate effects on *T*_m_ values, while the replacement of LNAs in the wing region with **5ara-T** or **5ara-^Me^C** resulted in thermal destabilization. These results are consistent with previous studies showing that the thermal stability of **5ara** modification is comparable to that of natural DNA but lower than that of LNA [27]. Furthermore, the *T*_m_ value of KN5ara-9 was similar to the addition of KN5ara-7 and KN5ara-8, indicating that the continuous introduction of modified analogs might not have an additional impact on thermal stability. The gapmers with fewer PS linkages (KN5ara-10 and KN5ara-11) were more stable than the corresponding full PS gapmers (KN5ara-3 and KN5ara-6). 

### 2.3. The Ability for E. coli RNase H1 Activation

**Figure 2:**
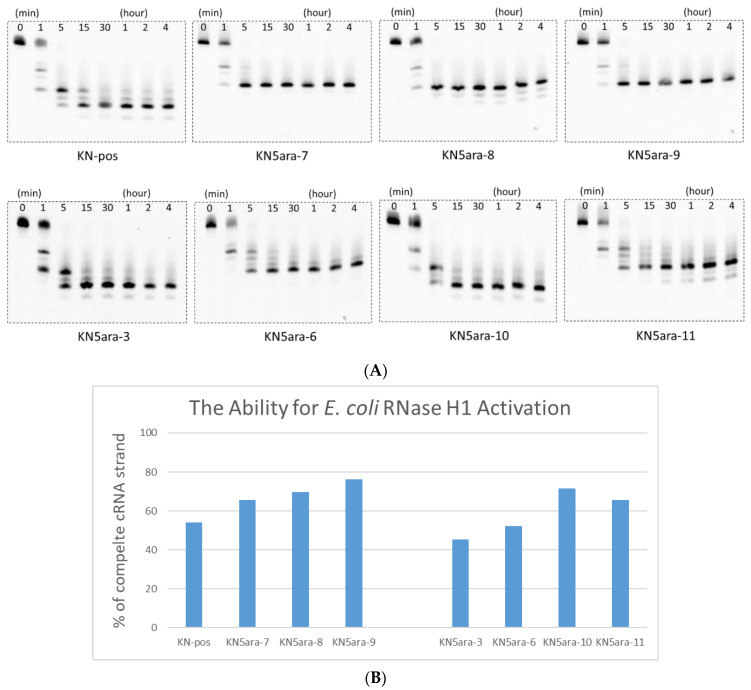
PAGE analysis of ASO/RNA duplexes treated in a buffer containing 50 mM Tris–HCl (pH 8.0), 75 mM KCl, 3 mM MgCl_2_, 10 mM dithiothreitol, and RNase H1 from E. coli. Sequence of complementary RNA strand (cRNA-2) is shown in Table S1. All duplexes were incubated in a buffer containing 50 mM Tris–HCl (pH 8.0), 75 mM KCl, 3 mM MgCl_2_, and 10 mM dithiothreitol, and then diluted RNase H1 solution (60 unit/L in H_2_O) was added; subsequently, the mixture was incubated at 37 °C for the required time. The reaction mixtures at various incubation times (0, 1, 5, 15, and 30 min and 1, 2, and 4 h) were analyzed with 20% PAGE and quantified with a luminescent image analyzer LAS-4000 (Fujifilm). (**A**) PAGE images obtained from luminescent analysis. (**B**) Percentage of complete cRNA strands remaining at 1 min, analyzed with ImageJ.2.4. Nuclease Resistance.

Before the in vitro cell experiments, we established a simple enzymatic reaction system using RNase H1 purified from *E. coli* to determine whether RNase H-mediated cleavage could be activated by KN5ara gapmers [33,34]. The KN5ara gapmers used in this experiment were mixed with fluorescein-labeled cRNA-2 at 1:5 before annealing. These duplexes were dissolved in a buffer containing 50 mM Tris–HCl (pH 8.0), 75 mM KCl, 3 mM MgCl_2_, and 10 mM dithiothreitol. Diluted *E. coli* RNase H1 solution (60 unit/L in H_2_O) was then added, and the mixture was incubated at 37 °C for the required time (0, 1, 5, 15, and 30 min and 1, 2, and 4 h). The aliquots were analyzed using 20% denaturing PAGE and then quantified using a luminescent image analyzer LAS-4000 (Fujifilm). As shown in Figure 2, even though the initial reaction velocity showed a slight change, it was confirmed that the complete cRNA strand was almost cleaved in KN5ara gapmers as well as in the positive control KN-pos after a 5 min reaction. Despite differences in each cleavage pattern, a single **5ara** modification moderately affected the cleavage mechanism of *E. coli* RNase H1, which was consistent with previous reports, because of the remaining recognition portions [27].

Notably, not all KN5ara gapmers were evaluated in this assay. Since the recognition of *E. coli* RNase H1 would begin with a few nucleotides from the 5ʹ-terminal of ASO, KN5ara-1 and -2, in which the **5ara** modification was inserted into 5ʹ-gap region, it might activate *E. coli* RNase H1 similarly with KN-pos. Meanwhile, as there are no significant differences for KN5ara-6 and -7, KN5ara-4 and -5 were found to be similar to the others. The antisense activity of all KN5ara gapmers is directly evaluated in the following section.

**Figure 3:**
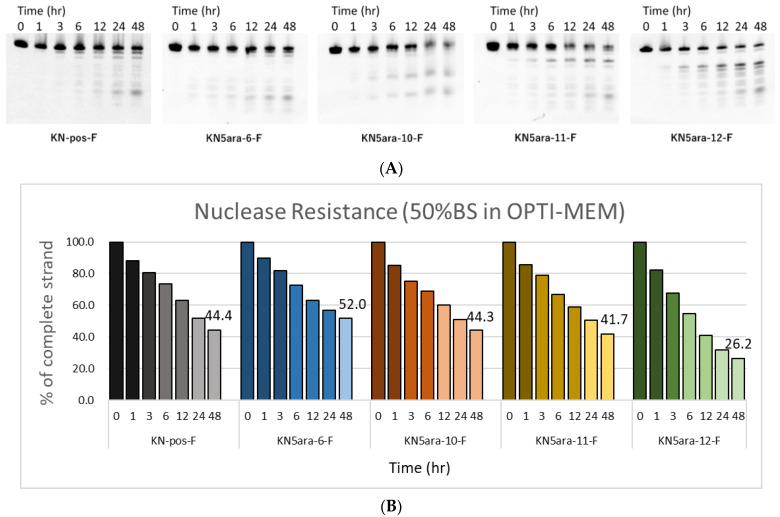
PAGE analysis of single-stranded KN5ara gapmers treated with OPTI-MEM containing 50% bovine serum (BS). Fluorescein-labeled KN5ara gapmers (300 pmol) were incubated in OPTI-MEM containing 50% BS. The reaction mixtures at various incubation time (0, 1, 3, 6, 12, 24, and 48 h) were analyzed by 20% PAGE containing 7 M urea and quantified by luminescent image analyzer LAS-4000 (Fujifilm). (**A**) PAGE images obtained from luminescent analysis. (**B**) Percentage of complete gapmer strands remaining at each time point, analyzed via ImageJ.

ASOs containing multiple **5ara** modifications showed extremely high enzyme tolerance in a 3% bovine serum (BS) experiment system [27]. LNAs and PS linkages are also expected to improve the stability of KN5ara gapmers during enzyme-mediated hydrolysis. However, PS linkages may lead to undesirable apoptosis because of their high affinity with plasma proteins, such as paraspeckle proteins [15]. We hypothesized that the application of the **5ara** modification could reduce the number of PS linkages while maintaining sufficient nuclease resistance. In this research, a series of fluorescein-labeled KN5ara gapmers were synthesized (Table S1), including positive control (KN-pos-F), and gapmers with full-PS (KN5ara-6-F) or with less PS linkages (KN5ara-10/11-F). For comparison, the gapmer without any **5ara** modification corresponding to KN5ara-11-F was prepared as well. The gapmers described above were dissolved in OPTI-MEM and incubated with 50% BS at 37 °C. During incubation, aliquots from the reactions were taken for the required time (0, 1, 3, 6, 12, 24 and 48 h), then analyzed with 20% PAGE containing 7 M urea and quantified using the Luminescent Image analyzer LAS-4000 (Fujifilm). The results are shown in Figure 3.

After 48 h incubation, approximately 44% of the KN-pos-F strands remained, while only 26% of KN5ara-12-F strands persisted, apparently owing to the decrease in the two PS linkages. A comparison of KN-pos-F and KN5ara-6-F showed that **5ara** modification could further increase the nuclease resistance of gapmers, resulting in a half-life of >48 h with 50% BS treatment. Meanwhile, 44% and 42% of the complete strands of KN5ara-10-F and KN5ara-11-F remained, respectively, indicating the same nuclease resistance as KN-pos-F. It is presumed that incorporating one **5ara** modification into the two PO linkages could reverse destabilization in enzymatic hydrolysis, which is caused by fewer PS linkages.

### 2.4. Antisense Activity

**Figure 4:**
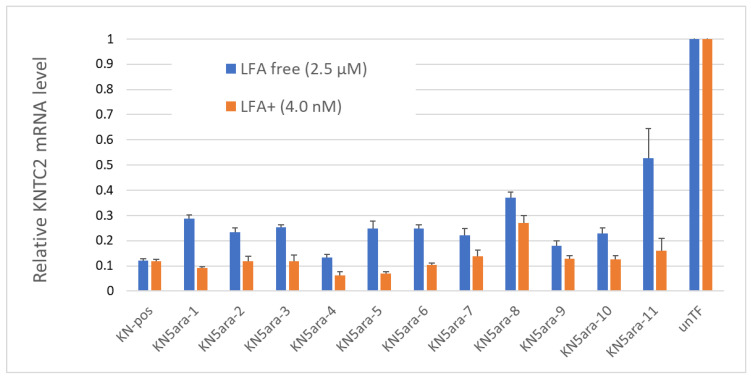
Relative *KNTC2* mRNA levels of each KN5ara gapmer. A549tGFP cells were treated with KN5ara gapmers at different final concentrations: 2.5 µM for lipofection-free group and 4.0 nM for 0.3% Lipofectamine^®^ 2000 (LFA) treated group. LFA-free groups were incubated with the gapmers for 48 h, and further incubated for 24 h after exchanging medium. LFA+ groups were incubated with the gapmers for 24 h, and further incubated for 24 h after exchanging medium. Treatment without gapmers was used as a control (unTF). The total mRNA inside cells were extracted, followed by the reverse transcription of the targeted *KNTC2* mRNA. Quantitative real-time polymerase chain reaction (qRT-PCR) was performed in duplicate, and the relative *KNTC2* mRNA levels were calculated via the ddCT method.

In addition to the physical experiments and simple in vitro enzymatic assays, in vitro cellular experiments were performed to knock down *KNTC2* in A549tGFP cells to evaluate the antisense activity of each KN5ara gapmer. The pre-cultured A549tGFP cells were treated with KN5ara gapmers at a final concentration of 4.0 nM or 2.5 µM, and were transfected with 0.3% Lipofectamine^®^ 2000 (LFA) or not, respectively. After incubation, total mRNA inside the cells was extracted, followed by reverse transcription of the targeted *KNTC2* mRNA. A quantitative real-time polymerase chain reaction (qRT-PCR) was performed in duplicate, and relative *KNTC2* mRNA levels were calculated, as shown in Figure 4.

The results suggested that there was no obvious change in the antisense activity of KN5ara gapmers with a single **5ara** modification when LFA was used. KN5ara-4/5 showed much higher *KNTC2* knockdown efficiency than the positive control KN-pos, although KN5ara-8 showed a 2-fold decrease. Interestingly, compared to KN5ara-8, KN5ara-9, which has another **5ara** modification at the same site as KN5ara-7, maintained antisense activity similar to that of KN5ara-7, suggesting that continuous **5ara** modifications may induce further improvement. Contrastingly, KN5ara-3 and KN5ara-10 exhibited similar antisense activity, whereas KN5ara-11 had slightly lower activity than KN5ara-6. 

In the LFA-free group, KN-pos showed antisense activity comparable to that in the LFA+ condition. All KN5ara gapmers were clearly less active, indicating that cellular uptake might be reduced, although the extent of the reduction varied greatly depending on the site of the **5ara** modification. Meanwhile, KN5ara-4 efficiently knocked down *KNTC2* mRNA, and the decrease in antisense activity of KN5ara-8 was also reversed by continuous **5ara** modifications (KN5ara-9). Notably, significant property degradation was observed in KN5ara-11, as compared to KN5ara-6, whereas KN5ara-10 showed similar or slightly better antisense activity than KN5ara-3. 

In more detail, it is obvious that KN5ara-4 showed the most effective antisense activity in both methods, when used with lipofection or not. The relative *KNTC2* mRNA level tended to increase according to the shift of **5ara** modification to the 3ʹ-terminal, which suggests **5ara** modification might be accepted well in the center of the gap region. Further investigation is need, however, of the synthesis of **5ara** modified adenosine and guanosine analogs. Moreover, according to previous studies, the incorporation of a single 2ʹ-OMe modification at gap position 2 could reduce the PS-derived toxicity while maintaining enough antisense activity [16]. In this study, KN5ara-3 and -10 obtained a **5ara** modification at gap position 2; additionally, KN5ara-10 retained even less antisense activity for PS linkages. Therefore, KN5ara-10 might be the most promising candidate for reducing PS-derived cytotoxicity. Further experiments studying the cytotoxicity are planned.

## 3. Conclusions

In summary, the novel synthesis of (*S*)-5ʹ-*C*-Aminopropyl-2ʹ-arabinofluoro-5-methyl-cytidine (**5ara-^Me^C**) was accomplished in this study. The properties of a series of LNA-wing antisense gapmers containing (*S*)-5ʹ-*C*-Aminopropyl-2ʹ-arabinofluoro (**5ara**) modification, named KN5ara gapmers, were evaluated. It was revealed that each KN5ara gapmer could bind to the complementary RNA strand with high affinity and could be thermally stable under biological condition. The ability for *E. coli* RNase H1 activation was moderately affected by the incorporation of a single **5ara** modification, although the cleavage pattern was obviously changed, which is consistent with previous reports. To determine whether the **5ara** modification could be an alternative to PS linkage, the nuclease resistance of several KN5ara gapmers was evaluated using a simple enzymatic reaction system. As a result, incorporating one **5ara** modification inside the two PO linkages could reverse the destabilization in enzymatic hydrolysis caused by fewer PS linkages. In addition, in vitro cellular experiments were performed to knockdown *KNTC2* in A549tGFP cells. The LFA+ group showed a relatively higher antisense activity than the LFA-free group, while KN5ara-4 showed superior antisense activity in both methods. Compared to KN5ara-7, KN5ara-8, and KN5ara-9, continuous **5ara** modifications at certain sites might induce further improvement. KN5ara-10, which contained fewer PS linkages, showed similar or slightly better antisense activity than the corresponding KN5ara-3. Hence, given the possibility of lower PS-derived cytotoxicity, KN5ara-10 might be the best candidate for KNTC2-targeted ASO for cancer therapy in this study. However, the further cytotoxicity assay of these KN5ara gapmers should be performed in future studies, and gapmers with fewer PS linkages than KN5ara-10 should also be synthesized and evaluated as well.

## 4. Experimental Sections

### 4.1. Solid-Phase Oligonucleotide Synthesis

The synthesis was carried out with a DNA/RNA synthesizer by the phosphoramidite method. After the synthesis, the RNA oligomers were cleaved from CPG beads and deprotected by treatment with concentrated NH_3_ solution/40% methylamine (1:1, *v*/*v*) for 10 min at 65 °C, while the DNA-based oligomers were treated with concentrated NH_3_ solution for 12 h at 55 °C. Notably, before the treatment of the NH_3_ solution, the CPG beads were treated with 10% dimethylamine in MeCN for 5 min followed by rinsing with MeCN to selectively remove cyanoethyl groups, if there are analogs introduced in oligomers. Then, 2ʹ-*O*-TBDMS groups in RNA oligomers were removed by Et_3_N∙3HF (125 µL) in DMSO (100 µL) for 1.5 h at 65 °C. The reaction was quenched with 0.1 M TEAA buffer (pH 7.0), and the mixture was desalted using a Sep-Pak C18 cartridge. The oligomers were purified by 20% PAGE containing 7 M urea to give highly purified oligonucleotides.

### 4.2. MALDI-TOF/MS Analysis of ONs

The spectra were obtained with a time-of-flight mass spectrometer equipped with a nitrogen laser (337 nm, 3 ns pulse). A solution of 3-hydroxypicolinic acid (3-HPA) and diammonium hydrogen citrate in H_2_O was used as a matrix. Data of synthetic ONs: KN-pos: *m/z* = 5346.49 (calcd for C_165_H_202_N_58_O_88_P_15_S_15_ [M-H]^−^, 5348.32); KNpos-F: *m/z* = 5884.62 (calcd for C_192_H_226_N_59_O_87_P_16_S_15_ [M-H]^−^, 5882.91); KN5ara-1: *m/z* = 5395.54 (calcd for C_167_H_208_N_59_O_87_FP_15_S_15_ [M-H]^−^, 5393.54); KN5ara-2: *m/z* = 5396.54 (calcd for C_167_H_207_N_59_O_87_FP_15_S_15_ [M-H]^−^, 5397.39); KN5ara-3: *m/z* = 5428.03 (calcd for C_168_H_208_N_59_O_88_FP_15_S_15_ [M-H]^−^, 5425.40); KN5ara-4: *m/z* = 5438.57 (calcd for C_169_H_210_N_59_O_88_FP_15_S_15_ [M-H]^−^, 5439.43); KN5ara-5: *m/z* = 5424.63 (calcd for C_168_H_208_N_59_O_88_FP_15_S_15_ [M-H]^−^, 5425.40); KN5ara-6: *m/z* = 5425.59 (calcd for C_168_H_208_N_59_O_88_FP_15_S_15_ [M-H]^−^, 5425.40); KN5ara-6-F: *m/z* = 5959.67 (calcd for C_195_H_232_N_60_O_97_FP_16_S_15_ [M-H]^−^, 5960.54); KN5ara-7: *m/z* = 5427.77 (calcd for C_168_H_208_N_59_O_88_FP_15_S_15_ [M-H]^−^, 5425.40); KN5ara-8: *m/z* = 5393.92 (calcd for C_167_H_208_N_59_O_87_FP_15_S_15_ [M-H]^−^, 5393.54); KN5ara-9: *m/z* = 5472.64 (calcd for C_170_H_214_N_60_O_87_F_2_P_15_S_15_ [M-H]^−^, 5468.59); KN5ara-10: *m/z* = 5392.62 (calcd for C_168_H_208_N_59_O_90_FP_15_S_13_ [M-H]^−^, 5389.59); KN5ara-10-F: *m/z* = 5927.72 (calcd for C_195_H_232_N_60_O_99_FP_16_S_13_ [M-H]^−^, 5928.59); KN5ara-11: *m/z* = 5391.73 (calcd for C_168_H_208_N_59_O_90_FP_15_S_13_ [M-H]^−^, 5389.59); KN5ara-11-F: *m/z* = 5927.72 (calcd for C_195_H_232_N_60_O_99_FP_16_S_13_ [M-H]^−^, 5928.94); KN5ara-12-F: *m/z* = 5852.67 (calcd for C_192_H_226_N_59_O_99_P_16_S_13_ [M-H]^−^, 5853.65); cRNA-1: *m/z* = 5046.38 (calcd for C_152_H_190_N_61_O_107_P_15_ [M-H]^−^, 5045.74); cRNA-2: *m/z* = 5585.96 (calcd for C_179_H_215_N_62_O_116_P_16_ [M-H]^−^, 5583.87).

### 4.3. Thermal Denaturation Study

The solution containing 3.0 µM ASO/RNA duplexes were prepared by mixing the ASOs (600 pmol) with complementary target **cRNA-1** (600 pmol) in a buffer of 10 mM sodium phosphate (pH 7.0) containing 100 mM NaCl, and then heated at 90–100 °C, followed by being cooled gradually to room temperature. Thermally induced transitions were monitored at 260 nm with a UV/vis spectrometer fitted with a temperature controller in quartz cuvettes with a path length of 1.0 cm. The sample temperature was increased by 0.5 °C/min. 

### 4.4. RNase H Assay

The ASO/RNA duplexes used for RNase H assay were prepared by mixing the ASOs (600 pmol) with fluorescein labeled complementary target **cRNA-2** (3000 pmol) in 75 µL of 50 mM Tris–HCl (pH 8.0) containing 75 mM KCl, 3 mM MgCl_2_ and 10 mM dithiothreitol, followed by heating at 90–100 °C for 5 min and cooling gradually to room temperature. Then, 70 µL diluted RNase H solution (60 unit/L in H_2_O) was added, and subsequently the mixture was incubated at 37 °C for the required time. Aliquots of 5 µL were diluted with 100% formamide (10 µL). Samples were subjected to electrophoresis in 20% PAGE containing 7M urea and quantified by Luminescent Image analyzer LAS-4000 (Fujifilm). 

### 4.5. Nuclease Resistance of Single-Stranded ASO

Fluorescein labeled ASOs (300 pmol) were dissolved in OPTI-MEM (37 µL) and used for the serum stability test. 1.0 µL of the oligomer solution was diluted in 10 µL stop solution (10% formamide in 10 mM EDTA) as the control sample (0 min). Then, 36 µL bovine serum was added to achieve a final concentration of 50% (*v*/*v*), and subsequently the mixture was incubated at 37 °C for the required time. Aliquots of 2.0 µL were diluted with 10 µL stop solution. Samples were subjected to electrophoresis in 20% PAGE containing 7M urea and quantified by Luminescent Image analyzer LAS-4000 (Fujifilm). 

### 4.6. ASOs In Vitro Activity Assay

A549tGFP cells were established by transfection with pGIPZ (Horizon Discovery) into a human lung cancer cell line A549 (ATCC). For LFA-free (LFA-) group, A549tGFP cells were plated into 96-well plates at 2 × 10^3^ cells/well, followed by the cultivation in D-MEM (Nissui) containing 10% fetal bovine serum (FBS), streptomycin (100 µg/mL) and penicillin (100 U/mL). Cells were incubated at 37 °C with 5% carbon dioxide for 24 h prior to the ASO administration. Then, the medium was exchanged into OPTI-MEM containing 2% FBS, and the *KNTC2*-targeting antisense gapmers were added into specified wells at the final concentration of 2.5 µM. After a 48-h incubation in 2% FBS_OPTI-MEM and a further 24-h incubation in 10% FBS_D-MEM, cells were retrieved, and the mRNAs were extracted using CellAmp Direct RNA Prep Kit for RT-PCR (Takara Bio). For LFA+ group, A549tGFP cells (ATCC) were plated into 96-well plates at 4 × 10^3^ cells/well and cultured in 10% FBS_D-MEM for 24 h prior to the ASO administration. After exchanging the medium into 10% FBS_OPTI-MEM, the *KNTC2*-targeting antisense gapmers, mixed with 0.3% Lipofectamine^®^ 2000 (Invitrogen) in advance, were added into specified wells at the final concentration of 4.0 nM and incubated for 24 h. Then, as the same as LFA- group, the medium was replaced by 10% FBS_D-MEM and cells were incubated for further 24 h, followed by the extraction of mRNAs. Treatment without ASOs was used as a control, named “unTF”.

Quantitative real-time polymerase chain reaction (qRT-PCR) of the targeted *KNTC2* mRNA was performed using One Step TB Green PrimeScript RT-PCR kit II (Takara), and Thermal Cycler Dice Real Time System (Takara). Each PCR reaction was performed in duplicate, and the relative *KNTC2* mRNA levels were calculated with the ddCT method, using beta-actin (ACTB) as a reference. The sequences of primers used in the qRT-PCR for Human *KNTC2* are 5ʹ-CCTCTCCATGCAGGAGTTAAGA-3ʹ for the forward primer, 5ʹ-GGTCTCGGGTCCTTGATTTTCT-3ʹ for the reverse primer. For Human ACTB, the sequences of forward primer and reverse primer are 5ʹ-GGAGCAATGATCTTGATCTT-3ʹ and 5ʹ-CCTTCCTGGGCATGGAGTCCT-3ʹ, respectively.

## Figures and Tables

**Table 1:** Sequence of each gapmer and *T*_m_ values of duplexes containing these gapmers.

Abbreviation of Gapmers	Sequence *^a,b^*	*T*_m_*^c^* (°C)	Δ*T*_m_ *^d^* (°C)
KN-pos	5′-T•A•^Me^C•d(A•T•G•G•A•G•C•T•T•T)•T•G•G-3′	64.8	—
KN5ara-1	5′-**T**•A•^Me^C•d(A•T•G•G•A•G•C•T•T•T)•T•G•G-3′	64.2	−0.5
KN5ara-2	5′-T•A•^**Me**^**C**•d(A•T•G•G•A•G•C•T•T•T)•T•G•G-3′	58.8	−6.0
KN5ara-3	5′-T•A•^Me^C•d(A•**T**• G•G•A•G•C•T•T•T)•T•G•G-3′	63.2	−1.6
KN5ara-4	5′-T•A•^Me^C•d(A•T•G•G•A•G•^**Me**^**C**•T•T•T)•T•G•G-3′	66.8	+2.0
KN5ara-5	5′-T•A•^Me^C•d(A•T•G•G•A•G•C•**T**•T•T)•T•G•G-3′	64.1	−0.7
KN5ara-6	5′-T•A•^Me^C•d(A•T•G•G•A•G•C•T•**T**•T)•T•G•G-3′	64.9	+0.1
KN5ara-7	5′-T•A•^Me^C•d(A•T•G•G•A•G•C•T•T•**T**)•T•G•G-3′	66.0	+1.2
KN5ara-8	5′-T•A•^Me^C•d(A•T•G•G•A•G•C•T•T•T)•**T**•G•G-3′	61.3	−3.5
KN5ara-9	5′-T•A•^Me^C•d(A•T•G•G•A•G•C•T•T•**T**)•**T**•G•G-3′	62.5	−2.2
KN5ara-10	5′-T•A•^Me^C•d(A**T**G•G•A•G•C•T•T•T)•T•G•G-3′	63.6	−1.2
KN5ara-11	5′-T•A•^Me^C•d(A•T•G•G•A•G•C•T**T**T)•T•G•G-3′	66.1	+1.4

*^a^* T and ^Me^C in red denote (*S*)-5ʹ-*C*-Aminopropyl-2ʹ-arabinofluoro-thymidine (**5ara-T**) and (*S*)-5ʹ-*C*-Aminopropyl-2ʹ-arabinofluoro-5-methyl-cytidine (**5ara-^Me^C**), respectively. The underlined ACGT denotes the corresponding LNAs. The black dots denote phosphorothioate linkages. *^b^* The sequence of the complementary RNA strand (cRNA-1) is shown in Table S1. *^c^* The *T*_m_ values were measured in 10 mM sodium phosphate buffer (pH 7.0) containing 100 mM NaCl. The concentration of the duplexes was 3 µM. All measurements were performed three times, and the data are shown as the average values. *^d^* Δ*T*_m_ represents [*T*_m_ (duplexes containing KN5ara-1–11) − *T*_m_ (duplex containing KN-pos)].

## Data Availability

All data involved in this paper was shown in the text or “Supplementary Materials”.

## Supplementary Materials

The following supporting information can be downloaded at: https://www.mdpi.com/article/10.3390/molecules27217384/s1, Scheme S1: Novel synthesis of (S)-5ʹ-C-aminopropyl-2ʹ-arabinofluoro-5-methyl-cytidine phosphoramidites, and General remark and details of the synthesis of compounds 2-4; Figure S1: Thermal Stability of Duplexes: the UV melting profiles of duplex composed of each KN5ara gapmer and cRNA-1; Table S1. The sequences of all oligonucleotides used in this research; ^1^H-, ^13^C- and ^19^F-NMR spectra of compounds 2-4 and ^31^P-NMR spectra of compound 4.

## Sample Availability

Samples of the compounds 1-4, KN-pos, KN5ara 1-11 (including the fluorescein labeled ones) and cRNA 1-2 are available from the authors.
