# Supplementary material for: Antisense Gapmers with LNA-Wings and (S)-5′-C-Aminopropyl-2′-arabinofluoro-nucleosides Could Efficiently Suppress the Expression of KNTC2
Source: Molecules. 2022 Oct 30;27(21):7384. doi: 10.3390/molecules27217384 (PMC9654562; doi:10.3390/molecules27217384)

Supplementary materials

Antisense gapmers with LNA-wings and (*S*)-5′-*C*-aminopropyl-2′-arabinofluoro-nucleosides could efficiently suppress the expression of *KNTC2*

Yujun Zhou[a], Shuichi Sakamoto[e] and Yoshihito Ueno[a, b, c, d]

[a]United Graduate School of Agricultural Science, [b]Faculty of Applied Biological Sciences, [c]Graduate School of Natural Science and Technology, and [d]Center for Highly Advanced Integration of Nano and Life Sciences (G-CHAIN), Gifu University, 1-1 Yanagido, Gifu, 501-1193, Japan.

[e]Institute of Microbial Chemistry (BIKAKEN) Numazu Branch, Microbial Chemistry Research Foundation, 18-24 Miyamoto, Numazu, Shizuoka 410-0301, Japan

*To whom correspondence should be addressed.

Phone: +81-58-293-2919; Fax: +81-58-293-2919.

E-mail: uenoy@gifu-u.ac.jp

Contents

Scheme S1. Novel synthesis of (*S*)-5′-*C*-aminopropyl-2′-arabinofluoro-5-methyl-cytidine phosphoramidites.

General remark and details of the synthesis of compounds **2-4**.

Figure S1. Thermal Stability of Duplexes: the UV melting profiles of duplex composed of each KN5ara gapmer and cRNA-1.

Table S1. The sequences of all oligonucleotides used in this research.

^{1}H-, ^{13}C- and ^{19}F-NMR spectra of compounds **2-4** and ^{31}P-NMR spectra of compound **4**.

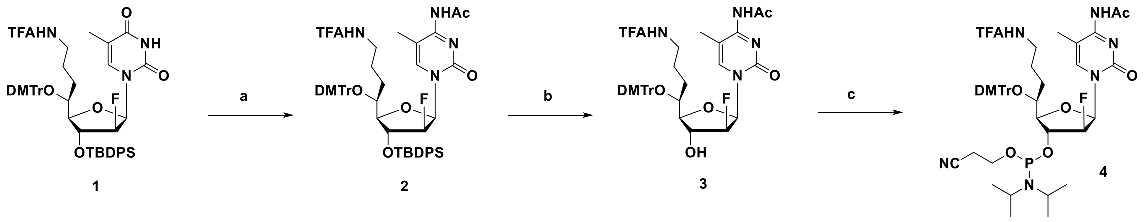

Scheme S1. Synthesis of (*S*)-5′-*C*-aminopropyl-2′-arabinofluoro-5-methyl-cytidine phosphoramidite **4**. [a]Reagents and conditions: (a)(i) TPSCl, DMAP, Et_3N, MeCN, r.t., 3 h; (ii) 28% NH_3 aq., r.t., 3 h; (iii) Ac_2O, pyridine, r.t., 2 h; **2**: 60%; (b) 1M TBAF-THF solution, THF, r.t., 3 h, **3**: 76%; (c) DIPEA, CEPCl, THF, r.t., 1.5 h, **4**: 74%.

General Remark.

All chemicals and dry solvents (DCE, DCM, DMF, MeCN, THF, and pyridine) were obtained from commercial sources and used without any further purification. Thin layer chromatography (TLC) was performed on silica gel plates precoated with fluorescent indicator with visualization by UV light or by dipping into a solution of 5% (v/v) concentrated H_2SO_4 in a mixture of *p*-anisaldehyde and methanol, and then heating. Silica gel (63-210 mesh) was used for column chromatography. ^{1}H NMR (400 or 500 M Hz), ^{13}C {1H} NMR (101 M Hz), ^{19}F NMR (376 M Hz), ^{31}P NMR (162 M Hz) were recorded on 400 or 500 M Hz NMR equipment. Acetonitrile-*d_3*, $CDCl_3$, or DMSO-*d_6* was used as a solvent for obtaining NMR spectra. Chemical shifts (δ) are given in parts per million (ppm) from $CDCl_3$ (7.26 ppm) for ^{1}H NMR spectra and $CDCl_3$ (77.2 ppm) for ^{13}C NMR spectra. The abbreviations s, d, t, q, and m signify singlet, doublet, triplet, quadruplet, and multiplet, respectively. High resolution mass spectra (HRMS) were obtained in positive ion electrospray ionization (ESI-TOF) mode.

***N*-Acetyl-2'-arabinofluoro-(*S*)-5'-*O*-[bis(4-methoxyphenyl)phenylmethyl]- 3'-*O*-[(1,1-dimethylethyl)diphenylsilyl]- 5-methyl-5'-*C*-trifluoroacetylaminopropyl-cytidine (2).** 4-Dimethylaminopyridine (0.10 g, 0.80 mmol), triethylamine (0.36 mL, 2.58 mmol), 2,4,6-triisopropylbenzenesulfonyl chloride (0.31 g, 1.04 mmol) were added into the solution of compound **1** in MeCN (5.0 mL) under argon atmosphere. The reactant was stirred for 3 h at room temperature, and then the 28% aqueous ammonia solution (2.08 mL) was dropwised. The mixture was stirred for more 3 h at room temperature and extracted with ethyl acetate and water; the organic layer was washed with brine, dried by Na_2SO_4, filtered and concentrated. The residue was dissolved with pyridine (5.0 mL) and acetic anhydride (0.10 mL, 1.04 mmol) was added. After being stirred for 2 h at room temperature, the reactant was extracted with ethyl acetate and water; the organic layer was washed with brine, dried by Na_2SO_4, filtered and concentrated. The crude material was purified by column chromatography (50% ethyl acetate in hexane) to afford desired product **2** (0.31 g, 60%). ^{1}H NMR (400 M Hz, $CDCl_3$) δ: 0.73-0.77 (m, 1H), 0.93 (s, 9H), 1.15-1.19 (m, 2H), 1.35-1.43 (m, 1H), 1.78 (s, 3H), 2.28 (s, 3H), 2.76-2.79 (m, 2H), 3.08 (d, *J* = 9.6 Hz, 1H), 3.71 (s, 3H), 3.72 (s, 3H), 4.10 (d, *J* = 3.2 Hz, 1H), 4.66 (dd, *J* = 4.6, 25.6 Hz, 1H), 5.24 (dd, *J* = 4.1, 53.2 Hz, 1H), 6.24 (dd, *J* = 4.1, 17.4 Hz, 1H), 6.73 (dd, *J* = 9.2, 17.8 Hz, 4H), 7.03-7.55 (m, 19H), 7.91 (s, 1H), 9.24 (t, *J* = 5.7 Hz, 1H), 9.91 (s, 1H); ^{13}C{^{1}H} NMR (101 M Hz, $CDCl_3$) δ: 13.80, 14.10, 24.27, 24.95, 26.49, 28.09, 54.97, 55.00, 72.54, 76.28, 76.55, 79.18, 83.87, 84.25, 85.60, 94.69, 96.59, 105.34, 111.58, 112.91, 114.45, 117.32, 126.62, 127.53, 127.87, 129.67, 129.80, 130.21, 131.62, 131.78, 135.12, 135.20, 135.80, 136.24, 142.05, 146.12, 153.50, 155.76, 156.11, 156.42, 158.03, 158.10, 162.76, 170.63; ^{19}F NMR (376 M Hz, $CDCl_3$) δ: -121.88 (d, *J* =73.76 Hz); HRMS (ESI-TOF) *m/z* Calcd for $C_{54}H_{58}F_4N_4NaO_8Si$ $[M + Na]^+$, 994.4000, found 994.3989.

***N*-Acetyl-2'-arabinofluoro-(*S*)-5'-*O*-[bis(4-methoxyphenyl)phenylmethyl]-5-methyl-5'-*C*-trifluoroacetylaminopropyl-cytidine (3).** Tetrabutylammonium fluoride (TBAF, 1.45 mL of a 1M solution in THF) was added in a solution of compound **2** (0.51 g, 0.68 mmol) in THF (14.5 mL) under argon atmosphere, and the reactant was stirred for 3 h at room temperature. The reactant was extracted with ethyl acetate and saturated $NaHCO_3$; the organic layer was washed with brine, dried by Na_2SO_4, filtered and concentrated. The crude material was purified by column chromatography (83% ethyl acetate in hexane) to afford desired product **3** as a white solid (0.39 g, 76%). ^1H NMR (400 M Hz, DMSO-*d6*) δ: 1.05-1,34 (m, 4H), 1.85 (s, 3H), 2.28 (s, 3H), 2.81-2.83 (m, 2H), 3.37-4.40 (m, 1H), 3.86-3.88 (m, 1H), 4.29 (dd, *J* =5.6, 21.6 Hz, 1H), 5.09 (dd, *J* =2.4, 55.2 Hz, 1H), 5.87 (d, *J* =5.2 Hz, 1H), 6.09 (dd, *J* =4, 16.8 Hz, 1H), 6.84-6.89 (m, 4H), 7.19-7.35 (m, 7H), 7.46 (d, *J* =7.2 Hz, 2H), 7.73 (s, 1H), 9.25 (t, *J* =5.5 Hz, 1H), 9.91 (bs, 1H); $^{13}C\{^1H\}$ NMR (101 M Hz, $CDCl_3$) δ: 13.80, 13.95, 20.05, 23.98, 27.87, 55.01, 55.06, 72.45, 74.06, 79.18, 83.15, 85.94, 113.04, 126.69, 127.65, 127.83, 130.10, 136.30, 136.51, 146.38, 158.16; ^{19}F NMR (376 M Hz, $CDCl_3$) δ: -119.40 (s); HRMS (ESI-TOF) *m/z* Calcd for $C_{38}H_{40}F_4N_4NaO_8$ $[M + Na]^+$, 779.2680, found 779.2663.

***N*-Acetyl-2'-arabinofluoro-3'-*O*-[2-Cyanoethoxy(diisopropylamino)phosphino]-(*S*)-5'-*O*-[bis(4-methoxyphenyl)phenylmethyl]- 5-methyl-5'-*C*-trifluoroacetylaminopropyl-cytidine (4).** N,N-diisopropylethylamine (DIPEA, 0.45 mL, 2.55 mmol) and 2-Cyanoethyl N,N-diisopropylchlorophosphoramidite (CEPCl, 0.23 mL, 1.02 mmol) were added in a solution of compound **3** (0.39 g, 0.51 mmol) in

THF (4.00 mL) in turn under argon atmosphere. The reactant was stirred for 1.5 h at room temperature, and then extracted with ethyl acetate and saturated $NaHCO_3$; the organic layer was washed with brine, dried by Na_2SO_4, filtered and concentrated. The crude material was purified by column chromatography (78% ethyl acetate in hexane) to afford desired product **4** as a white solid (0.36 g, 74%). ^{19}F NMR (376 M Hz, $CDCl_3$) δ: -119.89 (d, J = 184.24 Hz); ^{31}P NMR (162 M Hz, $CDCl_3$) δ: 151.64, 151.96. HRMS (ESI-TOF) *m/z* Calcd for $C_{47}H_{57}F_4N_6NaO_9P$ $[M + Na]^+$, 956.3900, found 956.3917.

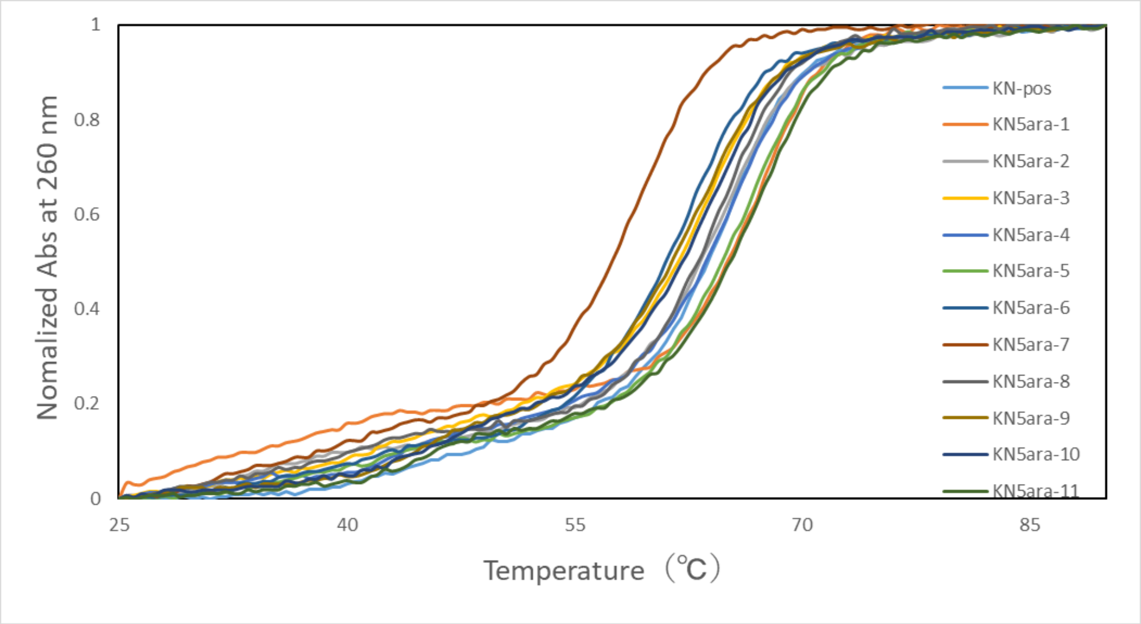

Figure S1. Thermal Stability of Duplexes: the UV melting profiles of duplex composed of each KN5ara gapmer and cRNA-1.

Table S1. The sequences of all oligonucleotides used in this research.

Abbreviation of oligonucleotides	Sequence [a]
KN-pos	5' - T▪A▪^{Me}C▪d(A▪T▪G▪G▪A▪G▪C▪T▪T▪T)▪T▪G▪G- 3'
KN5ara-1	5' - **T**▪A▪^{Me}C▪d(A▪T▪G▪G▪A▪G▪C▪T▪T▪T)▪T▪G▪G- 3'
KN5ara-2	5' - T▪A▪**^{Me}C**▪d(A▪T▪G▪G▪A▪G▪C▪T▪T▪T)▪T▪G▪G- 3'
KN5ara-3	5' - T▪A▪^{Me}C▪d(A▪**T**▪G▪G▪A▪G▪C▪T▪T▪T)▪T▪G▪G- 3'
KN5ara-4	5' - T▪A▪^{Me}C▪d(A▪T▪G▪G▪A▪G▪**^{Me}C**▪T▪T▪T)▪T▪G▪G- 3'
KN5ara-5	5' - T▪A▪^{Me}C▪d(A▪T▪G▪G▪A▪G▪C▪**T**▪T▪T)▪T▪G▪G- 3'
KN5ara-6	5' - T▪A▪^{Me}C▪d(A▪T▪G▪G▪A▪G▪C▪T▪**T**▪T)▪T▪G▪G- 3'
KN5ara-7	5' - T▪A▪^{Me}C▪d(A▪T▪G▪G▪A▪G▪C▪T▪T▪**T**)▪T▪G▪G- 3'
KN5ara-8	5' - T▪A▪^{Me}C▪d(A▪T▪G▪G▪A▪G▪C▪T▪T▪T)▪**T**▪G▪G- 3'
KN5ara-9	5' - T▪A▪^{Me}C▪d(A▪T▪G▪G▪A▪G▪C▪T▪T▪**T**)▪**T**▪G▪G- 3'
KN5ara-10	5' - T▪A▪^{Me}C▪d(A**T**G▪G▪A▪G▪C▪T▪T▪T)▪T▪G▪G- 3'
KN5ara-11	5' - T▪A▪^{Me}C▪d(A▪T▪G▪G▪A▪G▪C▪T**T**T)▪T▪G▪G- 3'
KN-pos-F	5' -F-T▪A▪^{Me}C▪d(A▪T▪G▪G▪A▪G▪C▪T▪T▪T)▪T▪G▪G- 3'
KN5ara-6-F	5' -F- T▪A▪^{Me}C▪d(A▪T▪G▪G▪A▪G▪C▪T▪**T**▪T)▪T▪G▪G - 3'
KN5ara-10-F	5' -F- T▪A▪^{Me}C▪d(A**T**G▪G▪A▪G▪C▪T▪T▪T)▪T▪G▪G - 3'
KN5ara-11-F	5' -F-▪A▪^{Me}C▪d(A▪T▪G▪G▪A▪G▪C▪T**T**T)▪T▪G▪G - 3'
KN5ara-12-F	5' -F-▪A▪^{Me}C▪d(A▪T▪G▪G▪A▪G▪C▪TTT)▪T▪G▪G - 3'
cRNA-1	5' - r(CCAAAAGCUCCAUGUA) - 3'
cRNA-2	5' -F- r(CCAAAAGCUCCAUGUA) - 3'

[a] T and ^{Me}C in red denote (*S*)-5'-*C*-Aminopropyl-2'-arabinofluoro-thymidine (**5ara-T**) and (*S*)-5'-*C*-Aminopropyl-2'-arabinofluoro-5-methyl-cytidine (**5ara-^{Me}C**), respectively. ACGT with underline denote the corresponding LNAs. Black dots denote the phosphorothioate (PS) linkages. F denotes fluorescein.

NMR spectra (^{1}H, ^{13}C, ^{19}F and ^{31}P)

^{1}H NMR spectrum of compound **2**

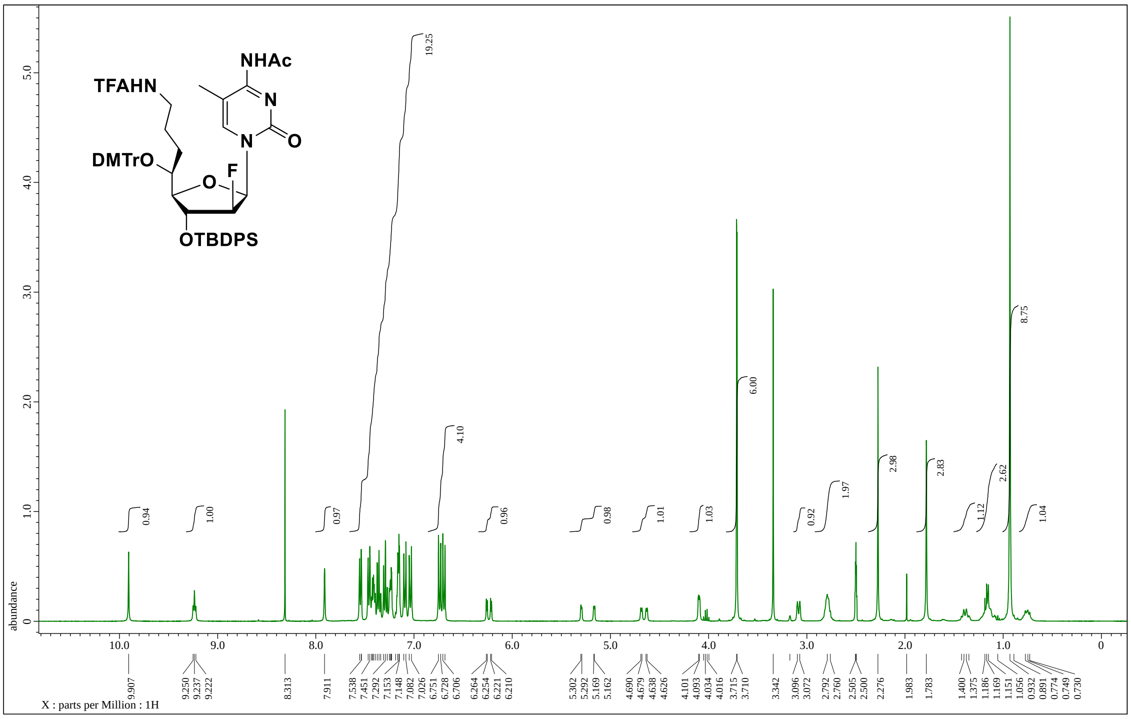

^{13}C NMR spectrum of compound **2**

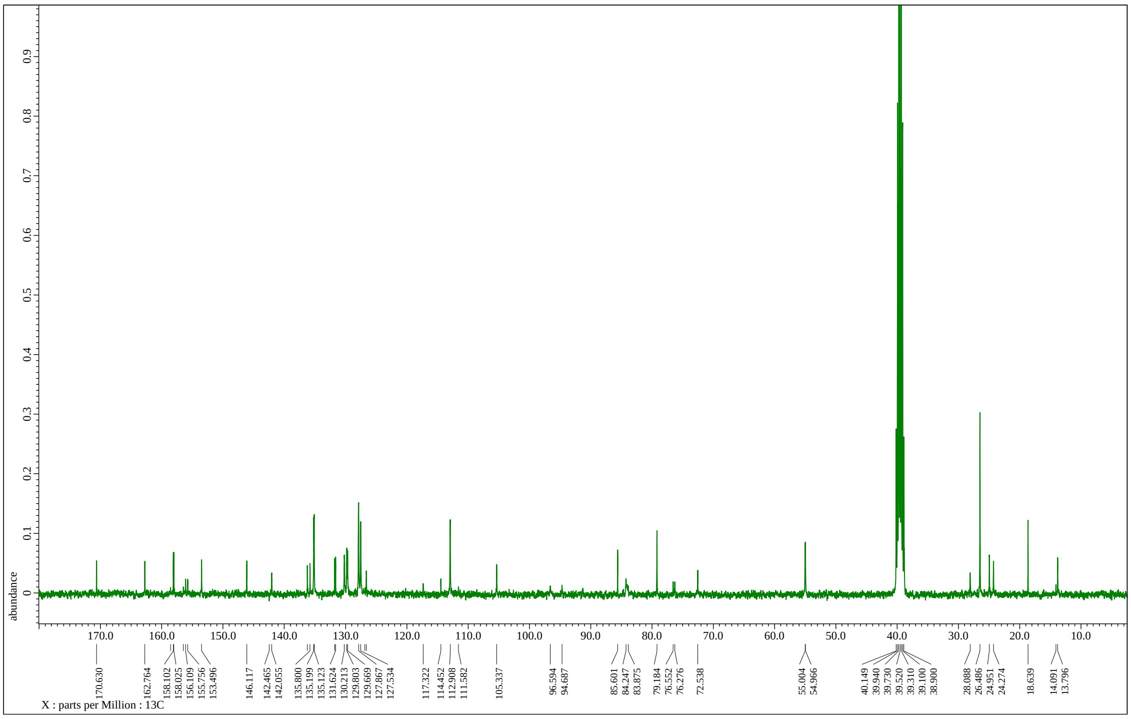

^{19}F NMR spectrum of compound **2**

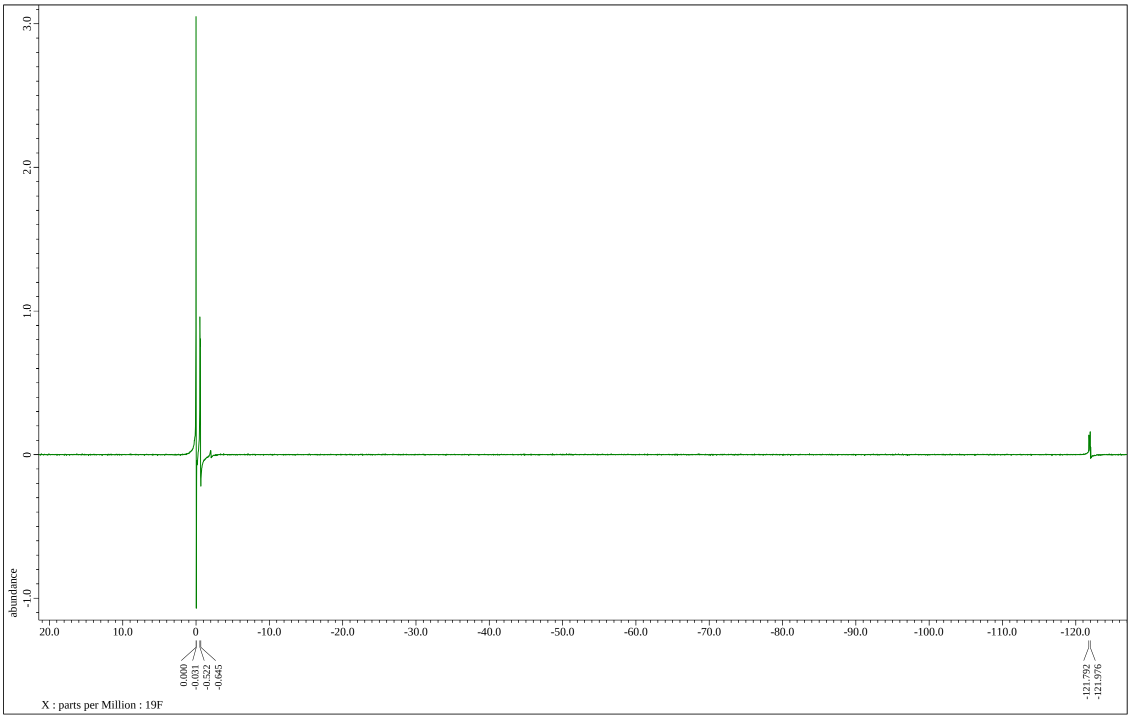

^{1}H NMR spectrum of compound **3**

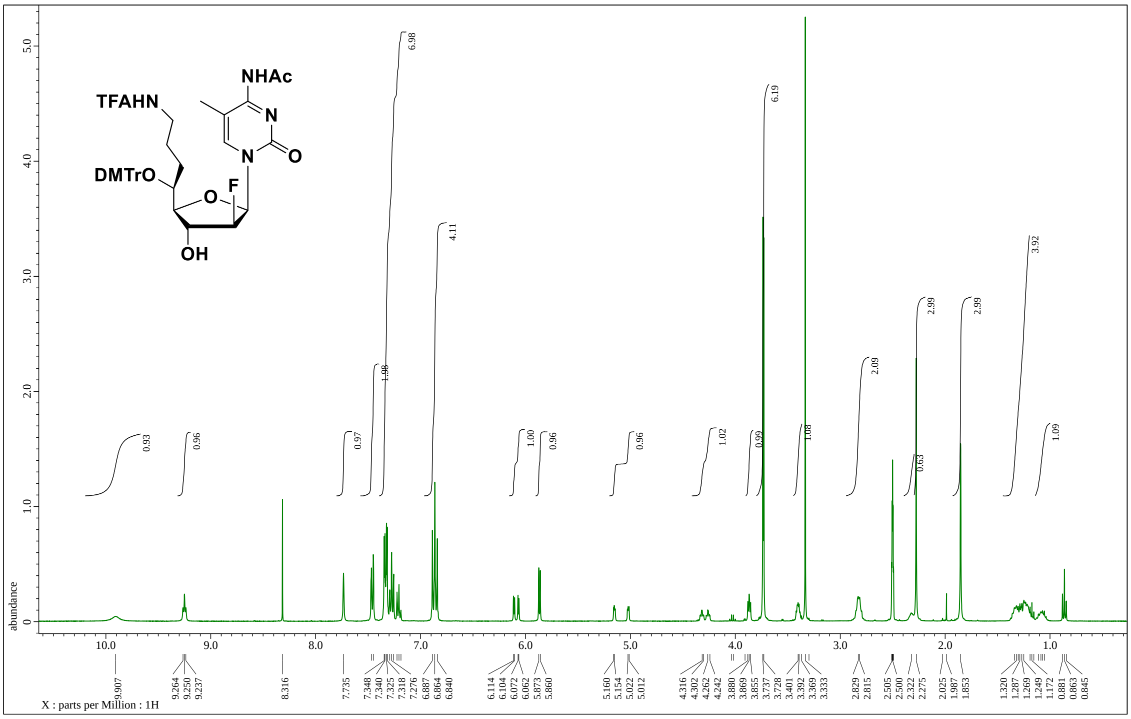

^{13}C NMR spectrum of compound **3**

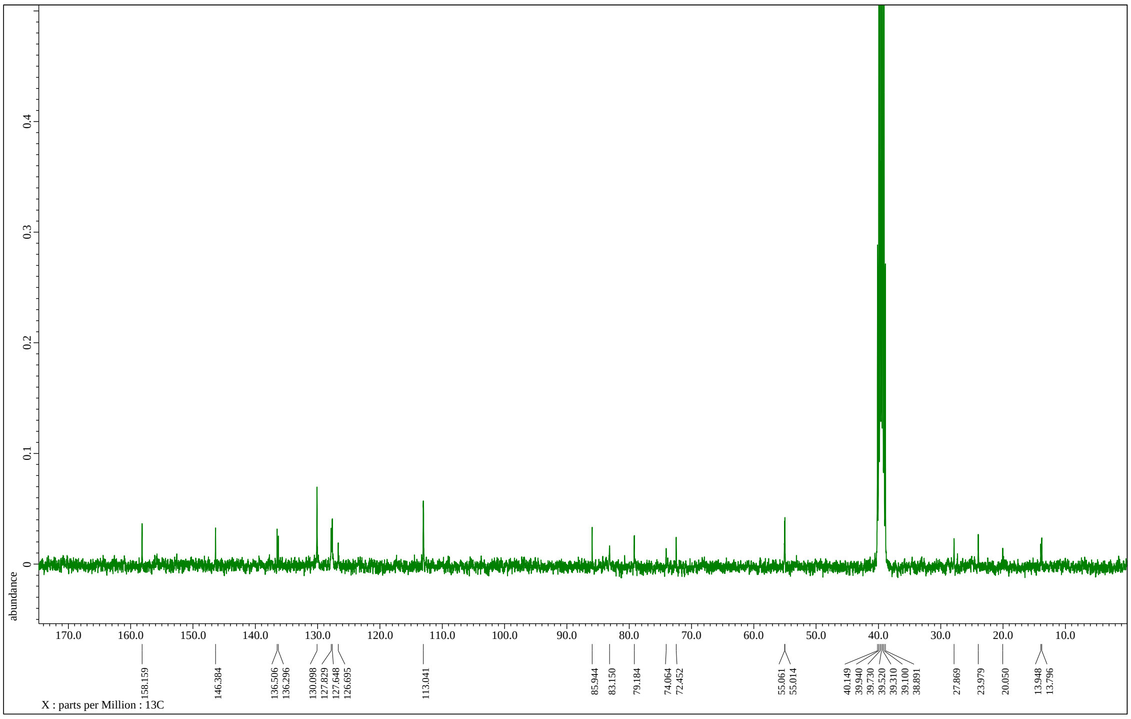

^{19}F NMR spectrum of compound **3**

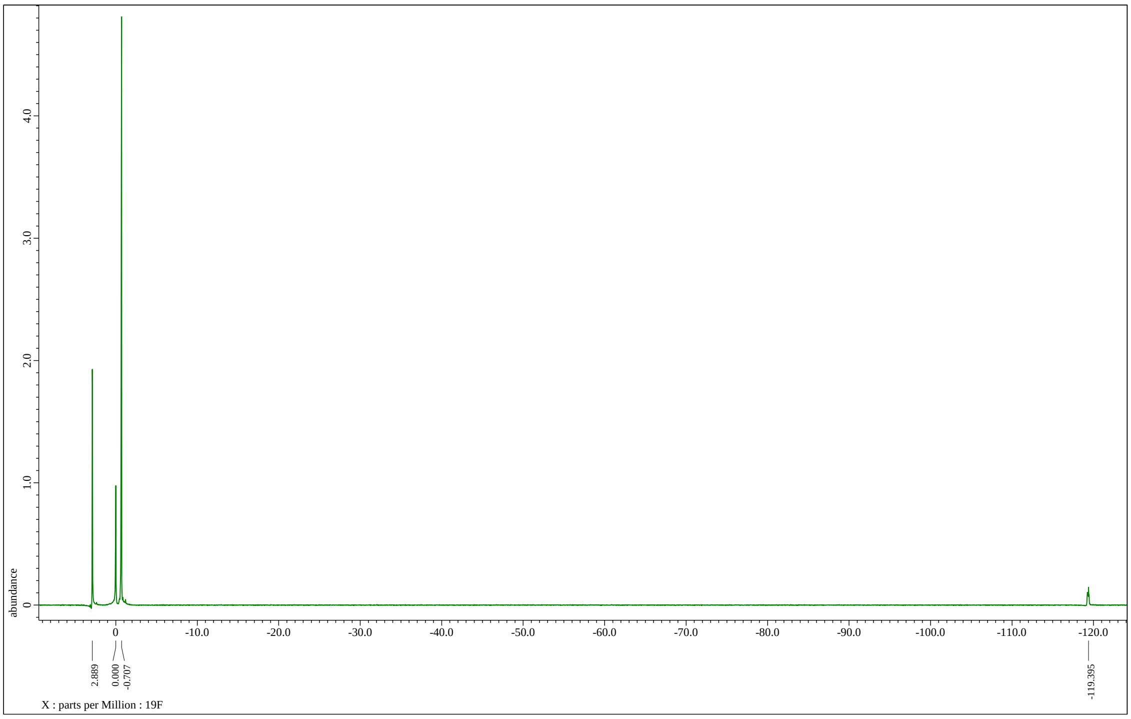

^{19}F NMR spectrum of compound **4**

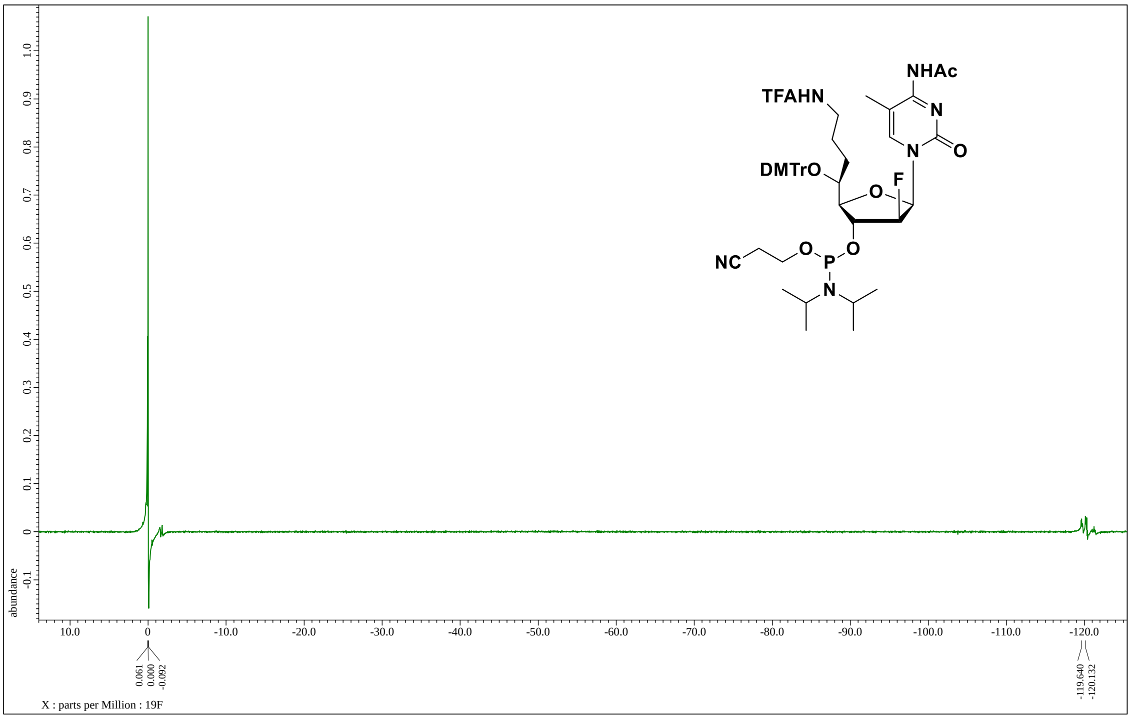

^{31}P NMR spectrum of compound **4**

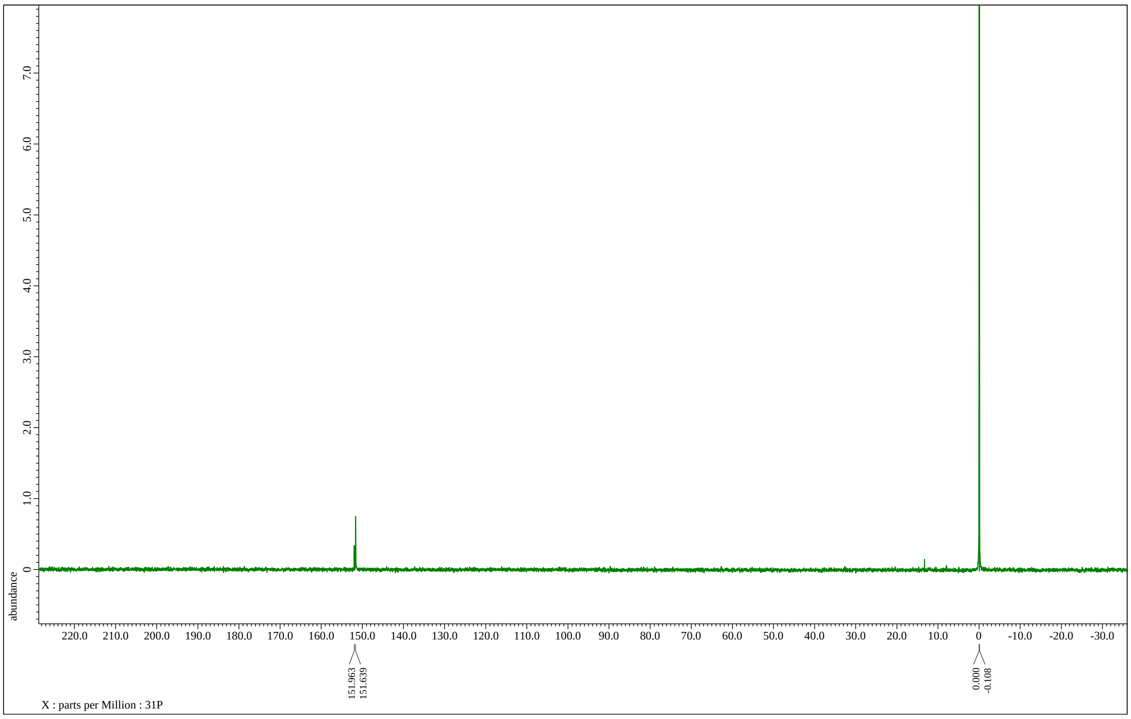

Supplement: Supplementary file 1 [file molecules-27-07384-s001.zip › molecules-1970198-supplementary.pdf]
